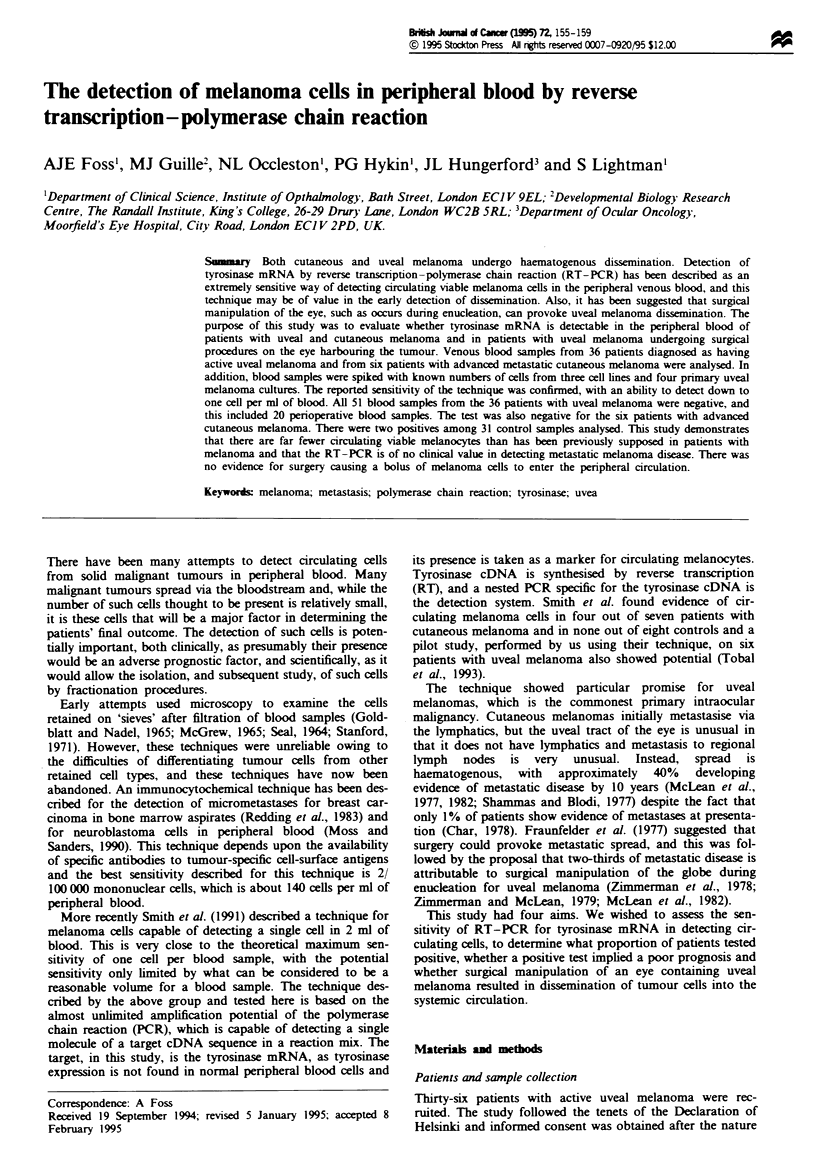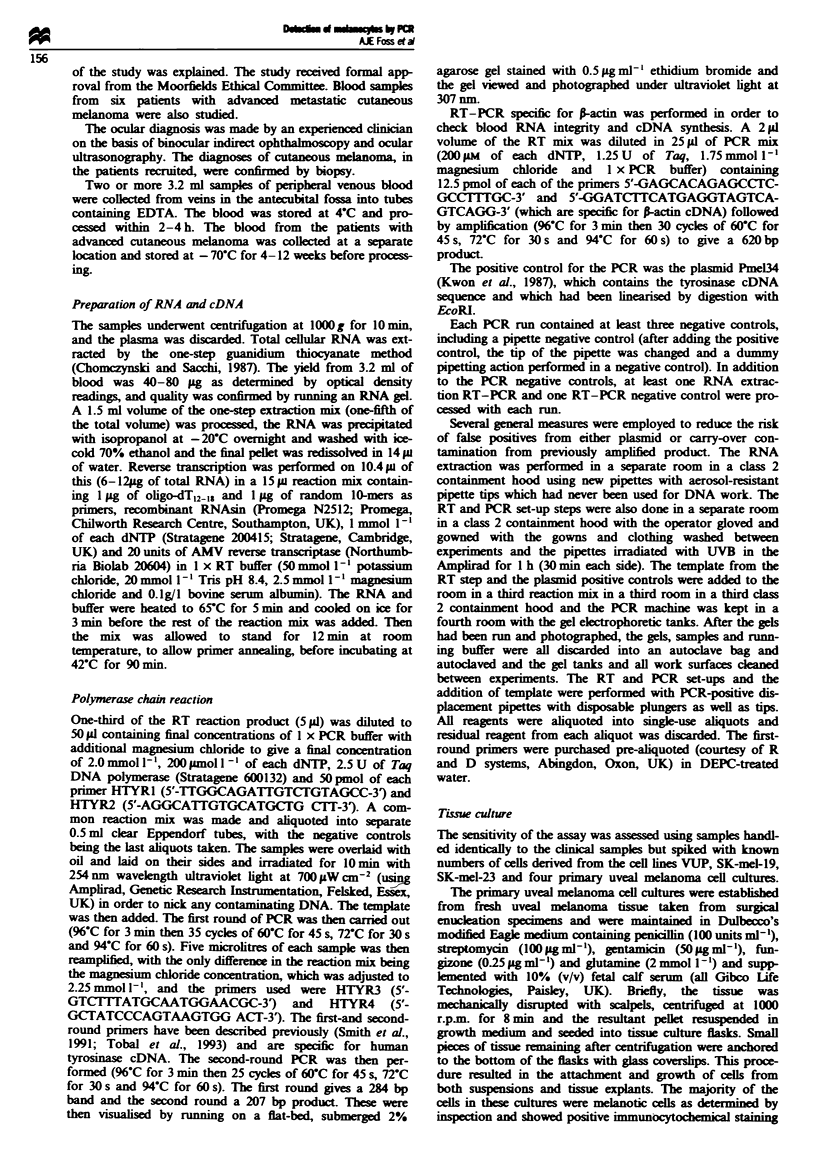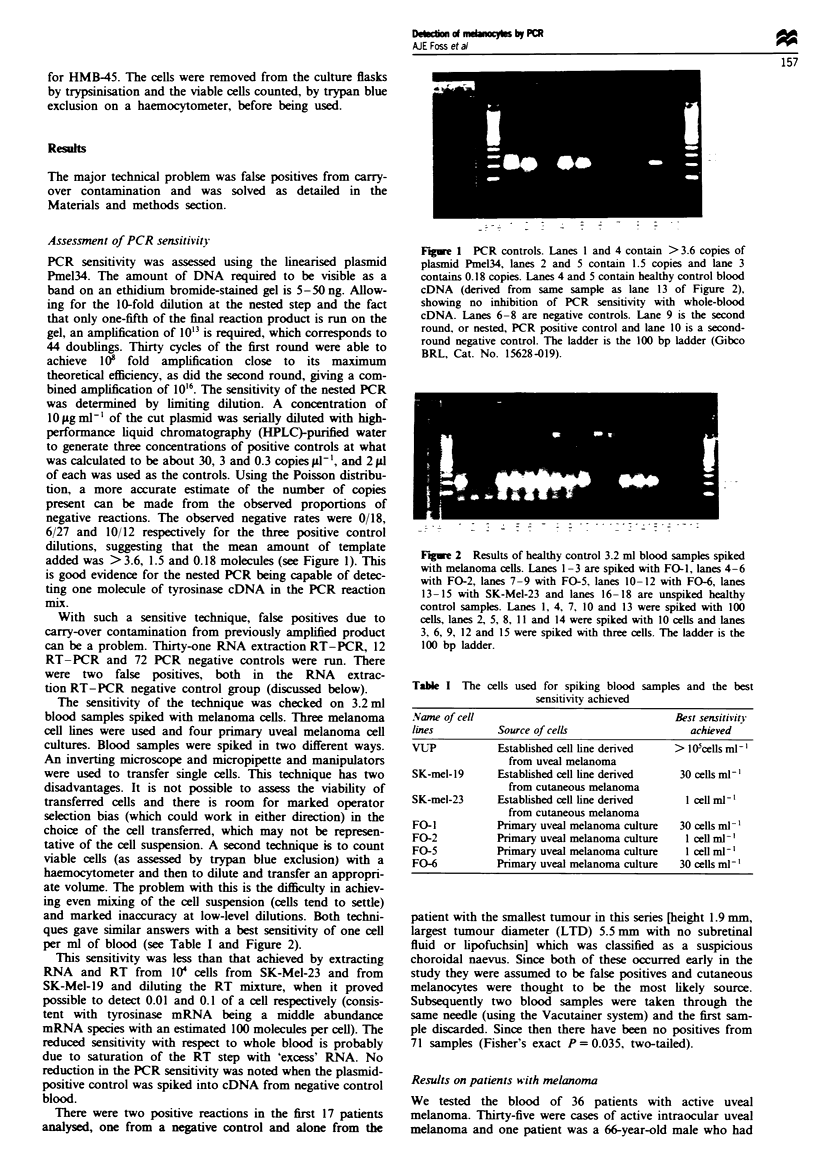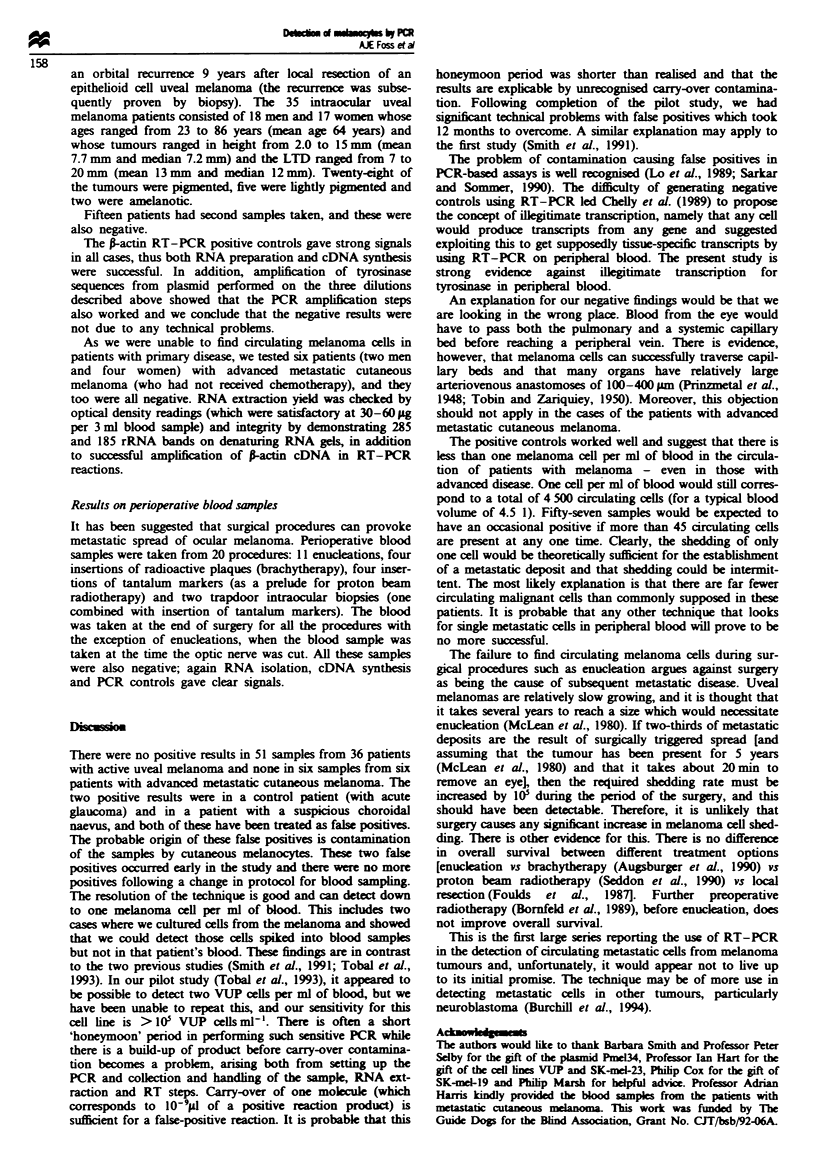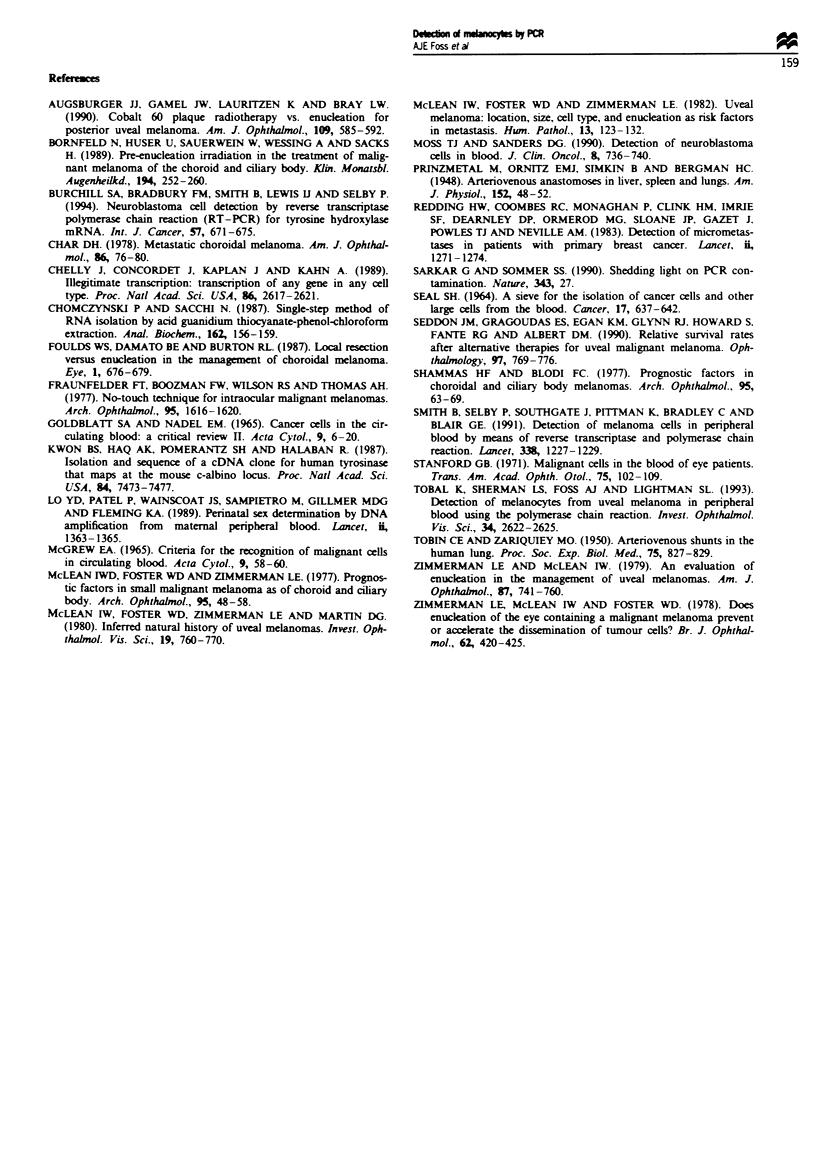# The detection of melanoma cells in peripheral blood by reverse transcription-polymerase chain reaction.

**DOI:** 10.1038/bjc.1995.293

**Published:** 1995-07

**Authors:** A. J. Foss, M. J. Guille, N. L. Occleston, P. G. Hykin, J. L. Hungerford, S. Lightman

**Affiliations:** Department of Clinical Science, Institute of Opthalmology, London, UK.

## Abstract

**Images:**


British Journal of Cancer (1995) 72, 155-159

© 1995 Stockton Press All rights reserved 0007-0920/95 $12.00

### The detection of melanoma cells in peripheral blood by reverse transcription-polymerase chain reaction

AJE Foss[1], MJ Guille[2], NL Occleston[1], PG Hykin[1], JL Hungerford[3] and S Lightman[1]

[1]*Department of Clinical Science, Institute of Opthalmology, Bath Street, London EC1V 9EL;* [2]*Developmental Biology Research Centre, The Randall Institute, King's College, 26-29 Drury Lane, London WC2B 5RL;* [3]*Department of Ocular Oncology, Moorfield's Eye Hospital, City Road, London EC1V 2PD, UK.*

**Summary** Both cutaneous and uveal melanoma undergo haematogenous dissemination. Detection of tyrosinase mRNA by reverse transcription-polymerase chain reaction (RT-PCR) has been described as an extremely sensitive way of detecting circulating viable melanoma cells in the peripheral venous blood, and this technique may be of value in the early detection of dissemination. Also, it has been suggested that surgical manipulation of the eye, such as occurs during enucleation, can provoke uveal melanoma dissemination. The purpose of this study was to evaluate whether tyrosinase mRNA is detectable in the peripheral blood of patients with uveal and cutaneous melanoma and in patients with uveal melanoma undergoing surgical procedures on the eye harbouring the tumour. Venous blood samples from 36 patients diagnosed as having active uveal melanoma and from six patients with advanced metastatic cutaneous melanoma were analysed. In addition, blood samples were spiked with known numbers of cells from three cell lines and four primary uveal melanoma cultures. The reported sensitivity of the technique was confirmed, with an ability to detect down to one cell per ml of blood. All 51 blood samples from the 36 patients with uveal melanoma were negative, and this included 20 perioperative blood samples. The test was also negative for the six patients with advanced cutaneous melanoma. There were two positives among 31 control samples analysed. This study demonstrates that there are far fewer circulating viable melanocytes than has been previously supposed in patients with melanoma and that the RT-PCR is of no clinical value in detecting metastatic melanoma disease. There was no evidence for surgery causing a bolus of melanoma cells to enter the peripheral circulation.

**Keywords:** melanoma; metastasis; polymerase chain reaction; tyrosinase; uvea

There have been many attempts to detect circulating cells from solid malignant tumours in peripheral blood. Many malignant tumours spread via the bloodstream and, while the number of such cells thought to be present is relatively small, it is these cells that will be a major factor in determining the patients' final outcome. The detection of such cells is potentially important, both clinically, as presumably their presence would be an adverse prognostic factor, and scientifically, as it would allow the isolation, and subsequent study, of such cells by fractionation procedures.

Early attempts used microscopy to examine the cells retained on 'sieves' after filtration of blood samples (Goldblatt and Nadel, 1965; McGrew, 1965; Seal, 1964; Stanford, 1971). However, these techniques were unreliable owing to the difficulties of differentiating tumour cells from other retained cell types, and these techniques have now been abandoned. An immunocytochemical technique has been described for the detection of micrometastases for breast carcinoma in bone marrow aspirates (Redding *et al.*, 1983) and for neuroblastoma cells in peripheral blood (Moss and Sanders, 1990). This technique depends upon the availability of specific antibodies to tumour-specific cell-surface antigens and the best sensitivity described for this technique is 2/100 000 mononuclear cells, which is about 140 cells per ml of peripheral blood.

More recently Smith *et al.* (1991) described a technique for melanoma cells capable of detecting a single cell in 2 ml of blood. This is very close to the theoretical maximum sensitivity of one cell per blood sample, with the potential sensitivity only limited by what can be considered to be a reasonable volume for a blood sample. The technique described by the above group and tested here is based on the almost unlimited amplification potential of the polymerase chain reaction (PCR), which is capable of detecting a single molecule of a target cDNA sequence in a reaction mix. The target, in this study, is the tyrosinase mRNA, as tyrosinase expression is not found in normal peripheral blood cells and its presence is taken as a marker for circulating melanocytes. Tyrosinase cDNA is synthesised by reverse transcription (RT), and a nested PCR specific for the tyrosinase cDNA is the detection system. Smith *et al.* found evidence of circulating melanoma cells in four out of seven patients with cutaneous melanoma and in none out of eight controls and a pilot study, performed by us using their technique, on six patients with uveal melanoma also showed potential (Tobal *et al.*, 1993).

The technique showed particular promise for uveal melanomas, which is the commonest primary intraocular malignancy. Cutaneous melanomas initially metastasise via the lymphatics, but the uveal tract of the eye is unusual in that it does not have lymphatics and metastasis to regional lymph nodes is very unusual. Instead, spread is haematogenous, with approximately 40% developing evidence of metastatic disease by 10 years (McLean *et al.*, 1977, 1982; Shammas and Blodi, 1977) despite the fact that only 1% of patients show evidence of metastases at presentation (Char, 1978). Fraunfelder *et al.* (1977) suggested that surgery could provoke metastatic spread, and this was followed by the proposal that two-thirds of metastatic disease is attributable to surgical manipulation of the globe during enucleation for uveal melanoma (Zimmerman *et al.*, 1978; Zimmerman and McLean, 1979; McLean *et al.*, 1982).

This study had four aims. We wished to assess the sensitivity of RT-PCR for tyrosinase mRNA in detecting circulating cells, to determine what proportion of patients tested positive, whether a positive test implied a poor prognosis and whether surgical manipulation of an eye containing uveal melanoma resulted in dissemination of tumour cells into the systemic circulation.

### Materials and methods

### *Patients and sample collection*

Thirty-six patients with active uveal melanoma were recruited. The study followed the tenets of the Declaration of Helsinki and informed consent was obtained after the nature

Correspondence: A Foss
Received 19 September 1994; revised 5 January 1995; accepted 8 February 1995

of the study was explained. The study received formal approval from the Moorfields Ethical Committee. Blood samples from six patients with advanced metastatic cutaneous melanoma were also studied.

The ocular diagnosis was made by an experienced clinician on the basis of binocular indirect ophthalmoscopy and ocular ultrasonography. The diagnoses of cutaneous melanoma, in the patients recruited, were confirmed by biopsy.

Two or more 3.2 ml samples of peripheral venous blood were collected from veins in the antecubital fossa into tubes containing EDTA. The blood was stored at 4°C and processed within 2–4 h. The blood from the patients with advanced cutaneous melanoma was collected at a separate location and stored at −70°C for 4–12 weeks before processing.

*Preparation of RNA and cDNA*

The samples underwent centrifugation at 1000 *g* for 10 min, and the plasma was discarded. Total cellular RNA was extracted by the one-step guanidium thiocyanate method (Chomczynski and Sacchi, 1987). The yield from 3.2 ml of blood was 40–80 μg as determined by optical density readings, and quality was confirmed by running an RNA gel. A 1.5 ml volume of the one-step extraction mix (one-fifth of the total volume) was processed, the RNA was precipitated with isopropanol at −20°C overnight and washed with ice-cold 70% ethanol and the final pellet was redissolved in 14 μl of water. Reverse transcription was performed on 10.4 μl of this (6–12μg of total RNA) in a 15 μl reaction mix containing 1 μg of oligo-d$T_{12-18}$ and 1 μg of random 10-mers as primers, recombinant RNAsin (Promega N2512; Promega, Chilworth Research Centre, Southampton, UK), 1 mmol $l^{-1}$ of each dNTP (Stratagene 200415; Stratagene, Cambridge, UK) and 20 units of AMV reverse transcriptase (Northumbria Biolab 20604) in 1 × RT buffer (50 mmol $l^{-1}$ potassium chloride, 20 mmol $l^{-1}$ Tris pH 8.4, 2.5 mmol $l^{-1}$ magnesium chloride and 0.1g/l bovine serum albumin). The RNA and buffer were heated to 65°C for 5 min and cooled on ice for 3 min before the rest of the reaction mix was added. Then the mix was allowed to stand for 12 min at room temperature, to allow primer annealing, before incubating at 42°C for 90 min.

*Polymerase chain reaction*

One-third of the RT reaction product (5 μl) was diluted to 50 μl containing final concentrations of 1 × PCR buffer with additional magnesium chloride to give a final concentration of 2.0 mmol $l^{-1}$, 200 μmol $l^{-1}$ of each dNTP, 2.5 U of *Taq* DNA polymerase (Stratagene 600132) and 50 pmol of each primer HTYR1 (5′-TTGGCAGATTGTCTGTAGCC-3′) and HTYR2 (5′-AGGCATTGTGCATGCTG CTT-3′). A common reaction mix was made and aliquoted into separate 0.5 ml clear Eppendorf tubes, with the negative controls being the last aliquots taken. The samples were overlaid with oil and laid on their sides and irradiated for 10 min with 254 nm wavelength ultraviolet light at 700 μW $cm^{-2}$ (using Amplirad, Genetic Research Instrumentation, Felsked, Essex, UK) in order to nick any contaminating DNA. The template was then added. The first round of PCR was then carried out (96°C for 3 min then 35 cycles of 60°C for 45 s, 72°C for 30 s and 94°C for 60 s). Five microlitres of each sample was then reamplified, with the only difference in the reaction mix being the magnesium chloride concentration, which was adjusted to 2.25 mmol $l^{-1}$, and the primers used were HTYR3 (5′-GTCTTTATGCAATGGAACGC-3′) and HTYR4 (5′-GCTATCCCAGTAAGTGG ACT-3′). The first-and second-round primers have been described previously (Smith *et al.*, 1991; Tobal *et al.*, 1993) and are specific for human tyrosinase cDNA. The second-round PCR was then performed (96°C for 3 min then 25 cycles of 60°C for 45 s, 72°C for 30 s and 94°C for 60 s). The first round gives a 284 bp band and the second round a 207 bp product. These were then visualised by running on a flat-bed, submerged 2% agarose gel stained with 0.5 μg $ml^{-1}$ ethidium bromide and the gel viewed and photographed under ultraviolet light at 307 nm.

RT–PCR specific for β-actin was performed in order to check blood RNA integrity and cDNA synthesis. A 2 μl volume of the RT mix was diluted in 25 μl of PCR mix (200 μM of each dNTP, 1.25 U of *Taq*, 1.75 mmol $l^{-1}$ magnesium chloride and 1 × PCR buffer) containing 12.5 pmol of each of the primers 5′-GAGCACAGAGCCTCGCCTTTGC-3′ and 5′-GGATCTTCATGAGGTAGTCAGTCAGG-3′ (which are specific for β-actin cDNA) followed by amplification (96°C for 3 min then 30 cycles of 60°C for 45 s, 72°C for 30 s and 94°C for 60 s) to give a 620 bp product.

The positive control for the PCR was the plasmid Pmel34 (Kwon *et al.*, 1987), which contains the tyrosinase cDNA sequence and which had been linearised by digestion with *Eco*RI.

Each PCR run contained at least three negative controls, including a pipette negative control (after adding the positive control, the tip of the pipette was changed and a dummy pipetting action performed in a negative control). In addition to the PCR negative controls, at least one RNA extraction RT–PCR and one RT–PCR negative control were processed with each run.

Several general measures were employed to reduce the risk of false positives from either plasmid or carry-over contamination from previously amplified product. The RNA extraction was performed in a separate room in a class 2 containment hood using new pipettes with aerosol-resistant pipette tips which had never been used for DNA work. The RT and PCR set-up steps were also done in a separate room in a class 2 containment hood with the operator gloved and gowned with the gowns and clothing washed between experiments and the pipettes irradiated with UVB in the Amplirad for 1 h (30 min each side). The template from the RT step and the plasmid positive controls were added to the room in a third reaction mix in a third room in a third class 2 containment hood and the PCR machine was kept in a fourth room with the gel electrophoretic tanks. After the gels had been run and photographed, the gels, samples and running buffer were all discarded into an autoclave bag and autoclaved and the gel tanks and all work surfaces cleaned between experiments. The RT and PCR set-ups and the addition of template were performed with PCR-positive displacement pipettes with disposable plungers as well as tips. All reagents were aliquoted into single-use aliquots and residual reagent from each aliquot was discarded. The first-round primers were purchased pre-aliquoted (courtesy of R and D systems, Abingdon, Oxon, UK) in DEPC-treated water.

*Tissue culture*

The sensitivity of the assay was assessed using samples handled identically to the clinical samples but spiked with known numbers of cells derived from the cell lines VUP, SK-mel-19, SK-mel-23 and four primary uveal melanoma cell cultures.

The primary uveal melanoma cell cultures were established from fresh uveal melanoma tissue taken from surgical enucleation specimens and were maintained in Dulbecco's modified Eagle medium containing penicillin (100 units $ml^{-1}$), streptomycin (100 μg $ml^{-1}$), gentamicin (50 μg $ml^{-1}$), fungizone (0.25 μg $ml^{-1}$) and glutamine (2 mmol $l^{-1}$) and supplemented with 10% (v/v) fetal calf serum (all Gibco Life Technologies, Paisley, UK). Briefly, the tissue was mechanically disrupted with scalpels, centrifuged at 1000 r.p.m. for 8 min and the resultant pellet resuspended in growth medium and seeded into tissue culture flasks. Small pieces of tissue remaining after centrifugation were anchored to the bottom of the flasks with glass coverslips. This procedure resulted in the attachment and growth of cells from both suspensions and tissue explants. The majority of the cells in these cultures were melanotic cells as determined by inspection and showed positive immunocytochemical staining for HMB-45. The cells were removed from the culture flasks by trypsinisation and the viable cells counted, by trypan blue exclusion on a haemocytometer, before being used.

### Results

The major technical problem was false positives from carry-over contamination and was solved as detailed in the Materials and methods section.

### *Assessment of PCR sensitivity*

PCR sensitivity was assessed using the linearised plasmid Pmel34. The amount of DNA required to be visible as a band on an ethidium bromide-stained gel is 5–50 ng. Allowing for the 10-fold dilution at the nested step and the fact that only one-fifth of the final reaction product is run on the gel, an amplification of $10^{13}$ is required, which corresponds to 44 doublings. Thirty cycles of the first round were able to achieve $10^8$ fold amplification close to its maximum theoretical efficiency, as did the second round, giving a combined amplification of $10^{16}$. The sensitivity of the nested PCR was determined by limiting dilution. A concentration of $10\ \mu g\ ml^{-1}$ of the cut plasmid was serially diluted with high-performance liquid chromatography (HPLC)-purified water to generate three concentrations of positive controls at what was calculated to be about 30, 3 and 0.3 copies $\mu l^{-1}$, and $2\ \mu l$ of each was used as the controls. Using the Poisson distribution, a more accurate estimate of the number of copies present can be made from the observed proportions of negative reactions. The observed negative rates were 0/18, 6/27 and 10/12 respectively for the three positive control dilutions, suggesting that the mean amount of template added was >3.6, 1.5 and 0.18 molecules (see Figure 1). This is good evidence for the nested PCR being capable of detecting one molecule of tyrosinase cDNA in the PCR reaction mix.

With such a sensitive technique, false positives due to carry-over contamination from previously amplified product can be a problem. Thirty-one RNA extraction RT–PCR, 12 RT–PCR and 72 PCR negative controls were run. There were two false positives, both in the RNA extraction RT–PCR negative control group (discussed below).

The sensitivity of the technique was checked on 3.2 ml blood samples spiked with melanoma cells. Three melanoma cell lines were used and four primary uveal melanoma cell cultures. Blood samples were spiked in two different ways. An inverting microscope and micropipette and manipulators were used to transfer single cells. This technique has two disadvantages. It is not possible to assess the viability of transferred cells and there is room for marked operator selection bias (which could work in either direction) in the choice of the cell transferred, which may not be representative of the cell suspension. A second technique is to count viable cells (as assessed by trypan blue exclusion) with a haemocytometer and then to dilute and transfer an appropriate volume. The problem with this is the difficulty in achieving even mixing of the cell suspension (cells tend to settle) and marked inaccuracy at low-level dilutions. Both techniques gave similar answers with a best sensitivity of one cell per ml of blood (see Table I and Figure 2).

This sensitivity was less than that achieved by extracting RNA and RT from $10^4$ cells from SK-Mel-23 and from SK-Mel-19 and diluting the RT mixture, when it proved possible to detect 0.01 and 0.1 of a cell respectively (consistent with tyrosinase mRNA being a middle abundance mRNA species with an estimated 100 molecules per cell). The reduced sensitivity with respect to whole blood is probably due to saturation of the RT step with 'excess' RNA. No reduction in the PCR sensitivity was noted when the plasmid-positive control was spiked into cDNA from negative control blood.

There were two positive reactions in the first 17 patients analysed, one from a negative control and alone from the patient with the smallest tumour in this series [height 1.9 mm, largest tumour diameter (LTD) 5.5 mm with no subretinal fluid or lipofuchsin] which was classified as a suspicious choroidal naevus. Since both of these occurred early in the study they were assumed to be false positives and cutaneous melanocytes were thought to be the most likely source. Subsequently two blood samples were taken through the same needle (using the Vacutainer system) and the first sample discarded. Since then there have been no positives from 71 samples (Fisher's exact $P = 0.035$, two-tailed).

**Figure 1** PCR controls. Lanes 1 and 4 contain >3.6 copies of plasmid Pmel34, lanes 2 and 5 contain 1.5 copies and lane 3 contains 0.18 copies. Lanes 4 and 5 contain healthy control blood cDNA (derived from same sample as lane 13 of Figure 2), showing no inhibition of PCR sensitivity with whole-blood cDNA. Lanes 6–8 are negative controls. Lane 9 is the second round, or nested, PCR positive control and lane 10 is a second-round negative control. The ladder is the 100 bp ladder (Gibco BRL, Cat. No. 15628-019).

**Figure 2** Results of healthy control 3.2 ml blood samples spiked with melanoma cells. Lanes 1–3 are spiked with FO-1, lanes 4–6 with FO-2, lanes 7–9 with FO-5, lanes 10–12 with FO-6, lanes 13–15 with SK-Mel-23 and lanes 16–18 are unspiked healthy control samples. Lanes 1, 4, 7, 10 and 13 were spiked with 100 cells, lanes 2, 5, 8, 11 and 14 were spiked with 10 cells and lanes 3, 6, 9, 12 and 15 were spiked with three cells. The ladder is the 100 bp ladder.

**Table I** The cells used for spiking blood samples and the best sensitivity achieved

| *Name of cell lines* | *Source of cells* | *Best sensitivity achieved* |
|---|---|---|
| VUP | Established cell line derived from uveal melanoma | $>10^5$cells $ml^{-1}$ |
| SK-mel-19 | Established cell line derived from cutaneous melanoma | 30 cells $ml^{-1}$ |
| SK-mel-23 | Established cell line derived from cutaneous melanoma | 1 cell $ml^{-1}$ |
| FO-1 | Primary uveal melanoma culture | 30 cells $ml^{-1}$ |
| FO-2 | Primary uveal melanoma culture | 1 cell $ml^{-1}$ |
| FO-5 | Primary uveal melanoma culture | 1 cell $ml^{-1}$ |
| FO-6 | Primary uveal melanoma culture | 30 cells $ml^{-1}$ |

### *Results on patients with melanoma*

We tested the blood of 36 patients with active uveal melanoma. Thirty-five were cases of active intraocular uveal melanoma and one patient was a 66-year-old male who had an orbital recurrence 9 years after local resection of an epithelioid cell uveal melanoma (the recurrence was subsequently proven by biopsy). The 35 intraocular uveal melanoma patients consisted of 18 men and 17 women whose ages ranged from 23 to 86 years (mean age 64 years) and whose tumours ranged in height from 2.0 to 15 mm (mean 7.7 mm and median 7.2 mm) and the LTD ranged from 7 to 20 mm (mean 13 mm and median 12 mm). Twenty-eight of the tumours were pigmented, five were lightly pigmented and two were amelanotic.

Fifteen patients had second samples taken, and these were also negative.

The β-actin RT-PCR positive controls gave strong signals in all cases, thus both RNA preparation and cDNA synthesis were successful. In addition, amplification of tyrosinase sequences from plasmid performed on the three dilutions described above showed that the PCR amplification steps also worked and we conclude that the negative results were not due to any technical problems.

As we were unable to find circulating melanoma cells in patients with primary disease, we tested six patients (two men and four women) with advanced metastatic cutaneous melanoma (who had not received chemotherapy), and they too were all negative. RNA extraction yield was checked by optical density readings (which were satisfactory at 30-60 μg per 3 ml blood sample) and integrity by demonstrating 285 and 185 rRNA bands on denaturing RNA gels, in addition to successful amplification of β-actin cDNA in RT-PCR reactions.

*Results on perioperative blood samples*

It has been suggested that surgical procedures can provoke metastatic spread of ocular melanoma. Perioperative blood samples were taken from 20 procedures: 11 enucleations, four insertions of radioactive plaques (brachytherapy), four insertions of tantalum markers (as a prelude for proton beam radiotherapy) and two trapdoor intraocular biopsies (one combined with insertion of tantalum markers). The blood was taken at the end of surgery for all the procedures with the exception of enucleations, when the blood sample was taken at the time the optic nerve was cut. All these samples were also negative; again RNA isolation, cDNA synthesis and PCR controls gave clear signals.

### Discussion

There were no positive results in 51 samples from 36 patients with active uveal melanoma and none in six samples from six patients with advanced metastatic cutaneous melanoma. The two positive results were in a control patient (with acute glaucoma) and in a patient with a suspicious choroidal naevus, and both of these have been treated as false positives. The probable origin of these false positives is contamination of the samples by cutaneous melanocytes. These two false positives occurred early in the study and there were no more positives following a change in protocol for blood sampling. The resolution of the technique is good and can detect down to one melanoma cell per ml of blood. This includes two cases where we cultured cells from the melanoma and showed that we could detect those cells spiked into blood samples but not in that patient's blood. These findings are in contrast to the two previous studies (Smith *et al.*, 1991; Tobal *et al.*, 1993). In our pilot study (Tobal *et al.*, 1993), it appeared to be possible to detect two VUP cells per ml of blood, but we have been unable to repeat this, and our sensitivity for this cell line is $>10^5$ VUP cells $ml^{-1}$. There is often a short 'honeymoon' period in performing such sensitive PCR while there is a build-up of product before carry-over contamination becomes a problem, arising both from setting up the PCR and collection and handling of the sample, RNA extraction and RT steps. Carry-over of one molecule (which corresponds to $10^{-9}$μl of a positive reaction product) is sufficient for a false-positive reaction. It is probable that this honeymoon period was shorter than realised and that the results are explicable by unrecognised carry-over contamination. Following completion of the pilot study, we had significant technical problems with false positives which took 12 months to overcome. A similar explanation may apply to the first study (Smith *et al.*, 1991).

The problem of contamination causing false positives in PCR-based assays is well recognised (Lo *et al.*, 1989; Sarkar and Sommer, 1990). The difficulty of generating negative controls using RT-PCR led Chelly *et al.* (1989) to propose the concept of illegitimate transcription, namely that any cell would produce transcripts from any gene and suggested exploiting this to get supposedly tissue-specific transcripts by using RT-PCR on peripheral blood. The present study is strong evidence against illegitimate transcription for tyrosinase in peripheral blood.

An explanation for our negative findings would be that we are looking in the wrong place. Blood from the eye would have to pass both the pulmonary and a systemic capillary bed before reaching a peripheral vein. There is evidence, however, that melanoma cells can successfully traverse capillary beds and that many organs have relatively large arteriovenous anastomoses of 100-400 μm (Prinzmetal *et al.*, 1948; Tobin and Zariquiey, 1950). Moreover, this objection should not apply in the cases of the patients with advanced metastatic cutaneous melanoma.

The positive controls worked well and suggest that there is less than one melanoma cell per ml of blood in the circulation of patients with melanoma – even in those with advanced disease. One cell per ml of blood would still correspond to a total of 4 500 circulating cells (for a typical blood volume of 4.5 l). Fifty-seven samples would be expected to have an occasional positive if more than 45 circulating cells are present at any one time. Clearly, the shedding of only one cell would be theoretically sufficient for the establishment of a metastatic deposit and that shedding could be intermittent. The most likely explanation is that there are far fewer circulating malignant cells than commonly supposed in these patients. It is probable that any other technique that looks for single metastatic cells in peripheral blood will prove to be no more successful.

The failure to find circulating melanoma cells during surgical procedures such as enucleation argues against surgery as being the cause of subsequent metastatic disease. Uveal melanomas are relatively slow growing, and it is thought that it takes several years to reach a size which would necessitate enucleation (McLean *et al.*, 1980). If two-thirds of metastatic deposits are the result of surgically triggered spread [and assuming that the tumour has been present for 5 years (McLean *et al.*, 1980) and that it takes about 20 min to remove an eye], then the required shedding rate must be increased by $10^5$ during the period of the surgery, and this should have been detectable. Therefore, it is unlikely that surgery causes any significant increase in melanoma cell shedding. There is other evidence for this. There is no difference in overall survival between different treatment options [enucleation *vs* brachytherapy (Augsburger *et al.*, 1990) *vs* proton beam radiotherapy (Seddon *et al.*, 1990) *vs* local resection (Foulds *et al.*, 1987]. Further preoperative radiotherapy (Bornfeld *et al.*, 1989), before enucleation, does not improve overall survival.

This is the first large series reporting the use of RT-PCR in the detection of circulating metastatic cells from melanoma tumours and, unfortunately, it would appear not to live up to its initial promise. The technique may be of more use in detecting metastatic cells in other tumours, particularly neuroblastoma (Burchill *et al.*, 1994).

**Acknowledgements**

The authors would like to thank Barbara Smith and Professor Peter Selby for the gift of the plasmid Pmel34, Professor Ian Hart for the gift of the cell lines VUP and SK-mel-23, Philip Cox for the gift of SK-mel-19 and Philip Marsh for helpful advice. Professor Adrian Harris kindly provided the blood samples from the patients with metastatic cutaneous melanoma. This work was funded by The Guide Dogs for the Blind Association, Grant No. CJT/bsb/92-06A.

**References**

AUGSBURGER JJ, GAMEL JW, LAURITZEN K AND BRAY LW. (1990). Cobalt 60 plaque radiotherapy vs. enucleation for posterior uveal melanoma. *Am. J. Ophthalmol.*, **109**, 585–592.

BORNFELD N, HUSER U, SAUERWEIN W, WESSING A AND SACKS H. (1989). Pre-enucleation irradiation in the treatment of malignant melanoma of the choroid and ciliary body. *Klin. Monatsbl. Augenheilkd.*, **194**, 252–260.

BURCHILL SA, BRADBURY FM, SMITH B, LEWIS IJ AND SELBY P. (1994). Neuroblastoma cell detection by reverse transcriptase polymerase chain reaction (RT–PCR) for tyrosine hydroxylase mRNA. *Int. J. Cancer*, **57**, 671–675.

CHAR DH. (1978). Metastatic choroidal melanoma. *Am. J. Ophthalmol.*, **86**, 76–80.

CHELLY J, CONCORDET J, KAPLAN J AND KAHN A. (1989). Illegitimate transcription: transcription of any gene in any cell type. *Proc. Natl Acad. Sci. USA*, **86**, 2617–2621.

CHOMCZYNSKI P AND SACCHI N. (1987). Single-step method of RNA isolation by acid guanidium thiocyanate-phenol-chloroform extraction. *Anal. Biochem.*, **162**, 156–159.

FOULDS WS, DAMATO BE AND BURTON RL. (1987). Local resection versus enucleation in the management of choroidal melanoma. *Eye*, **1**, 676–679.

FRAUNFELDER FT, BOOZMAN FW, WILSON RS AND THOMAS AH. (1977). No-touch technique for intraocular malignant melanomas. *Arch. Ophthalmol.*, **95**, 1616–1620.

GOLDBLATT SA AND NADEL EM. (1965). Cancer cells in the circulating blood: a critical review II. *Acta Cytol.*, **9**, 6–20.

KWON BS, HAQ AK, POMERANTZ SH AND HALABAN R. (1987). Isolation and sequence of a cDNA clone for human tyrosinase that maps at the mouse c-albino locus. *Proc. Natl Acad. Sci. USA*, **84**, 7473–7477.

LO YD, PATEL P, WAINSCOAT JS, SAMPIETRO M, GILLMER MDG AND FLEMING KA. (1989). Perinatal sex determination by DNA amplification from maternal peripheral blood. *Lancet*, **ii**, 1363–1365.

McGREW EA. (1965). Criteria for the recognition of malignant cells in circulating blood. *Acta Cytol.*, **9**, 58–60.

McLEAN IWD, FOSTER WD AND ZIMMERMAN LE. (1977). Prognostic factors in small malignant melanoma as of choroid and ciliary body. *Arch. Ophthalmol.*, **95**, 48–58.

McLEAN IW, FOSTER WD, ZIMMERMAN LE AND MARTIN DG. (1980). Inferred natural history of uveal melanomas. *Invest. Ophthalmol. Vis. Sci.*, **19**, 760–770.

McLEAN IW, FOSTER WD AND ZIMMERMAN LE. (1982). Uveal melanoma: location, size, cell type, and enucleation as risk factors in metastasis. *Hum. Pathol.*, **13**, 123–132.

MOSS TJ AND SANDERS DG. (1990). Detection of neuroblastoma cells in blood. *J. Clin. Oncol.*, **8**, 736–740.

PRINZMETAL M, ORNITZ EMJ, SIMKIN B AND BERGMAN HC. (1948). Arteriovenous anastomoses in liver, spleen and lungs. *Am. J. Physiol.*, **152**, 48–52.

REDDING HW, COOMBES RC, MONAGHAN P, CLINK HM, IMRIE SF, DEARNLEY DP, ORMEROD MG, SLOANE JP, GAZET J, POWLES TJ AND NEVILLE AM. (1983). Detection of micrometastases in patients with primary breast cancer. *Lancet*, **ii**, 1271–1274.

SARKAR G AND SOMMER SS. (1990). Shedding light on PCR contamination. *Nature*, **343**, 27.

SEAL SH. (1964). A sieve for the isolation of cancer cells and other large cells from the blood. *Cancer*, **17**, 637–642.

SEDDON JM, GRAGOUDAS ES, EGAN KM, GLYNN RJ, HOWARD S, FANTE RG AND ALBERT DM. (1990). Relative survival rates after alternative therapies for uveal malignant melanoma. *Ophthalmology*, **97**, 769–776.

SHAMMAS HF AND BLODI FC. (1977). Prognostic factors in choroidal and ciliary body melanomas. *Arch. Ophthalmol.*, **95**, 63–69.

SMITH B, SELBY P, SOUTHGATE J, PITTMAN K, BRADLEY C AND BLAIR GE. (1991). Detection of melanoma cells in peripheral blood by means of reverse transcriptase and polymerase chain reaction. *Lancet*, **338**, 1227–1229.

STANFORD GB. (1971). Malignant cells in the blood of eye patients. *Trans. Am. Acad. Ophth. Otol.*, **75**, 102–109.

TOBAL K, SHERMAN LS, FOSS AJ AND LIGHTMAN SL. (1993). Detection of melanocytes from uveal melanoma in peripheral blood using the polymerase chain reaction. *Invest. Ophthalmol. Vis. Sci.*, **34**, 2622–2625.

TOBIN CE AND ZARIQUIEY MO. (1950). Arteriovenous shunts in the human lung. *Proc. Soc. Exp. Biol. Med.*, **75**, 827–829.

ZIMMERMAN LE AND McLEAN IW. (1979). An evaluation of enucleation in the management of uveal melanomas. *Am. J. Ophthalmol.*, **87**, 741–760.

ZIMMERMAN LE, McLEAN IW AND FOSTER WD. (1978). Does enucleation of the eye containing a malignant melanoma prevent or accelerate the dissemination of tumour cells? *Br. J. Ophthalmol.*, **62**, 420–425.